# Effects of diethylstilbestrol exposure during gestation on both maternal and offspring behavior

**DOI:** 10.3389/fnins.2015.00079

**Published:** 2015-03-16

**Authors:** Kazuya Tomihara, Takahiro Zoshiki, Sayaka Y. Kukita, Kanako Nakamura, Ayuko Isogawa, Sawako Ishibashi, Ayumi Tanaka, Ayaka S. Kuraoka, Saki Matsumoto

**Affiliations:** Department of Psychology, Faculty of Law, Economics, and Humanities, Kagoshima UniversityKagoshima, Japan

**Keywords:** endocrine disruptor, maternal behavior, estrogenic agents, developmental deficits, cross-fostering method

## Abstract

Endocrine disruption during gestation impairs the physical and behavioral development of offspring. However, it is unclear whether endocrine disruption also impairs maternal behavior and in turn further contributes to the developmental and behavioral dysfunction of offspring. We orally administered the synthetic non-steroidal estrogen diethylstilbestrol (DES) to pregnant female C57BL/6J mice from gestation day 11–17 and then investigated the maternal behavior of mothers. In addition, we examined the direct effects of *in utero* DES exposure and the indirect effects of aberrant maternal behavior on offspring using the cross-fostering method. In mothers, endocrine disruption during gestation decreased maternal behavior. In addition, endocrine disruption of foster mother influenced anxiety-related behavior and passive avoidance learning of pups regardless of their exposure *in utero*. The influence of DES exposure *in utero*, irrespective of exposure to the foster mother, was also shown in female offspring. These results demonstrate the risks of endocrine disruptors on both mother as well as offspring and suggest that developmental deficits may stem from both *in utero* toxicity and aberrant maternal care.

## Introduction

Many chemicals released into the environment can act as endocrine disruptors by mimicking the action of estrogen. Diethylstilbestrol (DES) is an active synthetic non-steroidal estrogen widely used as a model chemical to study the effects of estrogenic endocrine disruptors on both the physical and behavioral development of offspring. For instance, perinatal exposure to DES induced reproductive abnormalities such as reduced sperm count (Mclachlan et al., 1975) and lower weight of reproductive organs (Goyal et al., 2003) in male offspring. Female rats (Kubo et al., 2003) and guinea pigs (Hines et al., 1987) prenatally exposed to DES showed a lower lordosis quotient and a higher incidence of rejection in response to male mounting behavior compared to that of controls. In mice of both sexes, prenatal exposure to DES also increased the frequency of aggressive behavior toward conspecifics (Palanza et al., 1999a,b). These results suggest that estrogenic actions *in utero* are critical for both prenatal and postnatal development of reproductive organs and the brain, resulting in long-term effects on behavior.

Proper hormonal regulation during the perinatal period is important not only for the behavioral development of offspring but also for the maternal behavior. Many studies have demonstrated remarkable changes in the circulating levels of several hormones during gestation and the hormonal changes influence the expression of maternal behavior. In rodents, the onset of maternal behavior in pregnant females coincides with a sharp decrease in progesterone and an increase in estrogen and prolactin around parturition. In parturition, secretion of oxytocin stimulates uterine contractions. Forced changes of these hormones around parturition facilitate the onset of maternal behavior. Removal of the uterus and fetus at 16–19 days of gestation results in similar hormonal changes and the induction of maternal behavior in female rat (Rosenblatt and Siegel, 1975). Reproductive experience also induced neuronal and functional changes in several brain areas considered important for regulating maternal behavior such as the medial preoptic area (MPOA) (Keyser-Marcus et al., 2001), amygdala (Pessoa and Adolphs, 2010; Pare and Duvarci, 2012), and hippocampus (Pawluski and Galea, 2006). Factors associated with motherhood such as nursing and other interactions with offspring may also mediate behavioral and neurobiological changes that facilitate maternal care. However, hormonal treatments to sexually naïve ovariectomized female rats induced similar behavioral and neurobiological responses (Bridges, 1984; Bimonte and Denenberg, 1999; Kinsley et al., 2006; De Castilhos et al., 2008, 2010), indicating that changes in hormones during gestation, such as estrogen, progesterone, prolactin and oxytocin, are paramount for inducing the neuroplastic reorganization of the maternal brain, resulting in significant changes in behavior that in turn may improve maternal care. Therefore, we predict that endocrine disruption during pregnancy will result in deficient maternal behavior. However, there have been few studies on the effects of endocrine disruptors on the mother. Two studies reported decreased maternal behavior from perinatal exposure to bisphenol A, an estrogenic endocrine disruptor (Palanza et al., 2002; Kundakovic et al., 2013), but it is still not known if exposure to other estrogenic agents during pregnancy can suppress maternal behavior. It is clear that maternal care affects offspring development and behavior (Liu et al., 1997; Calatayud et al., 2004). If maternal behavior is also influenced by exposure to endocrine disruptors during gestation, it is crucial to distinguish influences on offspring due to prenatal estrogenic agent exposure from those due to aberrant maternal care. In other words, the changes of maternal behavior by exposure to DES may affect the behavioral development of pups independently of or interactively with their own *in utero* exposure of DES. Thus, the purpose of the present study is to examine the influences of gestational exposure to DES on maternal behavior and to investigate whether changes in maternal behavior impact offspring development.

In the present study, we used a low dose of DES (0.1 μg/day) to examine the consequences of gestational exposure. In mice, exposure to DES at this dose abolished sex differences in time spent in the light area of light–dark transition tests apparatus (Tomihara et al., 2006), reduced step-through latency in a passive avoidance learning retention trial in males (Kaitsuka et al., 2007) and increased CaMKII autophosphorylation and Ca^2+^-independent activity in the hippocampus and cortex of males (Kaitsuka et al., 2007). Thus, we chose this dose because of the reliably measureable effects on offspring behavior and neurophysiology and hypothesized that the effects of DES may partially be mediated by altered maternal behavior. To distinguish the influence of prenatal environment from that of rearing, we used the cross-fostering method, whereby some DES-exposed offspring were reared by vehicle-treated dams and some offspring of vehicle-treated dams were reared by DES-exposed dams. We then investigated the anxiety-related behavior and passive avoidance learning in offspring. These experiments demonstrate that endocrine disruption by DES during gestation disturbs maternal behavior, leading to aberrant behavior of in offspring.

## Materials and methods

We conducted 2 experiments in this study. In experiment 1, we examined the effects of DES exposure during gestation on subsequent maternal behavior on postpartum days 1–10. In experiment 2, we examined differences in anxiety-related behaviors of DES-exposed and unexposed offspring cross-fostered by DES-exposed or unexposed mothers to determine whether changes in maternal behavior induced by DES impact offspring behavioral development independent of prenatal DES exposure.

### General methods

#### Animals and treatments

Pregnant C57BL/6J Jcl (C57BL) mice between 3- and 6-months-old were obtained from CREA Japan, Inc. (Tokyo, Japan) on gestational day (GD) 6. Pregnant mice were individually housed in plastic cages (182 × 260 × 128 mm) and maintained under a 12-h light/dark cycle (0:00/12:00 h) at constant temperature (22 ± 2°C) with laboratory chow and water available *ad libitum* throughout all experiments. Animals were randomly divided into vehicle (OIL) control and DES-exposed groups. DES-exposed mice were orally administered 0.1 μg DES (Sigma-Aldrich, MO, USA) dissolved in 30 μl corn oil once a day from GD 11–17. Animals in the OIL-control group were administered 30 μl corn oil (vehicle) alone. Both vehicle and DES were delivered by a syringe and the tip of a needle inserted into the mouth rather than directly into the stomach to reduce stress. All mice were left undisturbed until the day of delivery (postnatal day 0 or PND0), which was confirmed by daily inspection of cages. On PDN1, to control the mother's cost of caring for offspring, we culled the litters to a maximum of six and adjusted the sex ratio of pups to 1:1 or as close as possible, except for experiment 2 in which cross-fostering was conducted. When the litter was lesser than 6, we did not cull them. Litters were weaned on PND21 and maintained on laboratory chow (CE-2, CLEA Japan, Inc., Tokyo) and water *ad libitum*. Male and female offspring were group-housed separately at 2-4/cage that was the same size as described above.

#### Experiment 1: effects of DES on maternal behavior

Twenty pregnant females were administered either DES or vehicle (OIL), of which 19 (DES-exposed: *n* = 9, OIL-control: *n* = 10) delivered a total of 110 offspring. The mean of the initial litter size and the sex ratio of female pups did not differ between groups (DES-exposed: 7.89 and 51.1%, OIL-control: 7.60 and 50.7%). We examined maternal behavior of the DES-exposed and OIL-control mice in two situations without observer intervention in the home cage as well as after handling and brief separation from pups. On PNDs 1, 3, 4, 5, 6, 7, 9, and 10, spontaneous maternal behavior in the home cage was assessed for 1 h at 1-min intervals by instantaneous sampling. The scored behaviors and their definitions were modified from those used in the studies by Palanza et al. (2002) and Fleming and Rosenblatt (1974): (a) Arched-back posture (female adopts a crouching posture with body arched over the pups), (b) Licking pups (licking or grooming pups), (c) Retrieving (picking up pups and transporting them), (d) Forced lactation (female is outside the nest engaged in another behavior but was reached by pups and suckles one or more), (e) Nest building (pushing or pulling nest material or picking up nest material in her mouth while inside or outside the nest), (f) In nest (inside the nest without exhibiting maternal behavior), (g) Eating/drinking (nibbling on a food pellet or drinking from the water bottle), (h) Self-grooming, (i) Resting (lying motionless outside the nest, not involved in any other form of behavior, and with no pups suckling), and (j) Locomotion (moving around the cage).

On PNDs 2 and 8, the pups were removed from the home cage for 10 min and then four randomly selected pups were placed one in each corner of the cage. Dams were videotaped for 30 min, and the following behaviors were coded using an event recorder: arched-back posture, licking pups, retrieving pups, forced lactation, and nest building. All observations were conducted under a dim red light during the dark phase (14:30-15:30).

#### Experiment 2: maternal effects on the behavior of adult offspring prenatally exposed to DES

We used the cross-fostering method to distinguish the effects of prenatal DES from the effects of maternal care. Twenty-two pregnant females (DES-exposed: *n* = 11, OIL-control: *n* = 11) and their 73 offspring were tested. The day after delivery, we culled the litters to control litter size (5–8) and sex ratio to nearly 1:1 and then conducted cross-fostering. The mean of the initial litter size and the sex ratio of female pups did not differ between groups (DES-exposed: 8.19 and 46.4%, OIL-control: 7.09 and 46.1%). The litters of five DES-exposed mothers were cross-fostered by other (DES mother–DES pups, referred to as the DES-des group). The other six litters of DES-exposed mothers were cross-fostered by vehicle-treated dams and six litters from vehicle-treated dams were cross-fostered by DES-exposed mothers (DES-oil and OIL-des groups, respectively). The remaining litters from vehicle-treated dams were cross-fostered by other vehicle-treated dams (OIL-oil group). The total numbers of pups in each group were as follows: DES-des, male *n* = 17, female *n* = 14; DES-oil, male *n* = 20, female *n* = 21; OIL-des: male *n* = 21, female *n* = 21; and OIL-oil: male *n* = 15, female *n* = 14. When the pups were weaned at PDN21, body weight and anogenital distance were measured. Starting on PDN60, the open-field (OF), elevated plus maze (EPM), and passive avoidance learning (PAL) tests were conducted consecutively on separate days always 2 h after lights were off.

The OF test was performed under a red dim light in a wooden test apparatus (600 × 600 × 250 mm) painted gray. The floor of the apparatus was equally divided into 16 areas (150 × 150 mm) by black lines. At the beginning of the test, a mouse was placed gently in a corner square with its head facing the corner. Animals were permitted to ambulate freely during the next 5 min and were videotaped by a camera attached approximately 100 cm above the apparatus. After the test, the number of transitions across area boundaries and time spent in the four central areas were recorded from video observation.

The EPM was made of gray-painted wood and consisted of a 60 × 60 mm center platform and four arms (60 × 300 mm) extending from the platform in a cross formation, with two opposing arms enclosed by side walls (300 × 150 mm) and two open arms. The entire maze was elevated 30 cm above the floor and illuminated from above by a dim red light. At the beginning of the test, a mouse was placed in the center platform facing one of the open arms and behavior was recorded for 5 min by a camera attached approximately 100 cm above the apparatus. The number of entries into and time spent in the open arms and closed arms were recorded from video observation. We analyzed the time in the open arms to total arm time (%) and the number of entries into these arms to total arm entries (%) as indices of anxiety-like behavior and the number of entries into the closed arms as an index of activity.

The PAL test apparatus was a rectangular Plexiglas chamber consisting of two compartments, one white and the other black, separated by a common wall (Takei Scientific Instruments Co., Ltd., Niigata, Japan). On day 1, a conditioning trial was conducted. Subjects were placed in the compartment with white walls, and the door into the compartment with black walls was opened 3 min later. After the mice entered the dark compartment, a 0.7 μA shock was applied to the floor of the dark chamber for 3 s. The subjects were returned to their home cages 1 min after shock delivery. The next day, subjects were placed individually in the light compartment for the test trials. Six seconds after introduction, the door was opened and their behavior was monitored for 180 s or until the subject crossed into the dark compartment. The latency (s) to cross was recorded for each trial.

### Statistical analyses

All data are presented as mean ± standard error (SE). Two-sample *t*-tests were used to compare means between DES and OIL-control groups. When the data did not fit a normal distribution, the Mann–Whitney *U*-test was used as an alternative. Two-Way ANOVAs were used when two factors were analyzed such as maternal and offspring exposure to DES. When necessary a test of a simple main effect was conducted to estimate the group difference at each level.

#### Ethics

All experimental procedures were in strict accordance with the guidelines of the Care and Use of Laboratory Animals in Kagoshima University and approved by the Ethics Committee for Animal Experimentation at Kagoshima University.

## Results

### Effects of DES on maternal behavior

DES exposure during gestation reduced subsequent maternal behavior by dams in the undisturbed condition. Mothers fed with 0.1 μg DES/day (DES group) showed decreased levels of arched-back posture [*U*_(10/9)_ = 13, *p* < 0.01] and increased resting [*t*_(17)_ = 2.89, *p* < 0.05] in the home cage compared to those of vehicle (corn oil)-treated dams (OIL group) (Table 1). Total time spent licking pups was also lower in the DES group, although the difference did not reach statistical significance [*t*_(17)_ = 2.09, *p* = 0.053]. In contrast, there were no significant differences in maternal behavior test scores between DES and OIL groups following brief separation from pups on PNDs 2 and 8 (Table 1).

**Table 1:** **Maternal behavior of DES-treated and OIL-treated (control) mothers**.

	**Home cage**		**After a brief separation from pups**			
			**Day 2**		**Day 8**	
	**OIL**	**DES**	**OIL**	**DES**	**OIL**	**DES**
Arched-back posture	18.0 (1.9)	11.0 (0.8)^**^	4.6 (3.1)	0.6 (0.6)	22.8 (10.5)	9.0 (7.4)
Licking pups	3.2 (0.3)	2.2 (0.3)	5.0 (0.8)	8.8 (1.0)	23.0 (5.5)	15.2 (3.5)
Retrieving	0.1 (0.0)	0.1 (0.0)	8.0 (1.5)	7.2 (1.0)	6.2 (0.8)	5.2 (1.2)
Forced lactation	3.9 (0.8)	4.4 (1.3)	0.0 (0.0)	0.0 (0.0)	13.8 (10.2)	10.0 (6.3)
Nest building	2.2 (0.4)	2.3 (0.7)	11.4 (7.2)	4.4 (2.5)	5.6 (3.6)	7.8 (3.2)
In nest	2.4 (0.2)	2.6 (0.5)	–	–	–	–
Eating/drinking	16.3 (1.5)	16.3 (1.2)	–	–	–	–
Self-grooming	1.8 (0.4)	2.7 (0.3)	–	–	–	–
Resting	3.5 (0.8)	8.9 (1.7)^*^	–	–	–	–
Locomotion	8.8 (1.1)	9.5 (1.1)	–	–	–	–

In the undisturbed home cage observations, values represent mean number (SE) of time bins in which the behavior was observed. In the test after a brief separation from pups, values of “Retrieving” represent mean number of instances of the behavior, and the rest of the values are time (s) spent performing the behavior. All values are converted to relative one per hour.

** p < 0.01,

* *p < 0.05 vs. OIL control*.

### Maternal effects on the behavior of adult offspring

The results of experiment 2 demonstrated that behavioral changes in offspring prenatally exposed to DES were, at least partially, due to the effects on maternal behavior independent of direct DES exposure *in utero*. Two-Way ANOVA (maternal exposure × offspring exposure) revealed a significant effect of maternal exposure to DES, regardless of offspring exposure (Table 2). In male offspring, DES exposure of the foster mother had a significant effect on open field activity as measured by the total number of transitions between areas [*F*_(1/69)_ = 6.52, *p* < 0.05]; specifically, male offspring exhibited more transitions (hyperactivity) when fostered by DES-exposed mothers than when fostered by oil-treated mothers (OIL-mother: 127.8 ± 4.2, DES-mother: 141.5 ± 4.6, means of pooled data by mother treatments). In female offspring, the time spent in the OF central area [*F*_(1/66)_ = 4.26, *p* < 0.05], the ratio of open arm entries in the EPM [*F*_(1/66)_ = 5.79, *p* < 0.05], and the latency to cross to the dark shock chamber in test trials of the PAL [*F*_(1/66)_ = 4.82, *p* < 0.05] differed depending on treatment of the foster mother. Specifically, time spent in the OF central area was shorter (OIL-mother: 73.8 ± 2.1 s, DES-mother: 68.6 ± 2.1 s), the ratio of open-arm entries was lower (OIL-mother: 20.2 ± 2.4%, DES-mother: 13.1 ± 2.1%), and latency to enter the shock compartment was longer (OIL-mother: 135.6 ± 11.3 s, DES-mother: 163.4 ± 8.0 s) in female offspring reared by DES-exposed foster mothers compared to females reared by oil-exposed foster mothers.

**Table 2:** **Body development and behavior of offspring prenatally exposed to DES or oil vehicle (OIL) and fostered by DES-treated or OIL-treated mothers**.

	**Treatment of foster mother**									***p*-values**					
		**Female offspring**				**Male offspring**				**Main effect of treatment**				**Interaction**	
		**OIL**		**DES**		**OIL**		**DES**		**Mother**		**Offspring**			
	**Treatment of pups**	**Oil**	**Des**	**Oil**	**Des**	**Oil**	**Des**	**Oil**	**Des**	**Female**	**Male**	**Female**	**Male**	**Female**	**Male**
	Body weight (mg)	8.7 (0.3)	8.7 (0.2)	8.8 (0.2)	8.7 (0.3)	8.3 (0.4)	9.0 (0.2)	9.3 (0.2)	8.9 (0.3)						
	AGD (mm)	4.4 (0.1)	4.4 (0.2)	4.4 (0.1)	4.5 (0.2)	7.4 (0.2)	8.1 (0.2)	7.8 (0.2)	7.8 (0.1)						
OF	Number of transitions	140.5 (6.9)	135.7 (5.2)	142.2 (8.2)	110.4 (5.6)	112.7 (6.1)	138.6 (4.5)	143.8 (6.1)	138.7 (7.0)		<0.05	<0.05			<0.05
	Time spent in center area (s)	74.4 (2.7)	73.3 (2.9)	72.0 (2.5)	63.5 (3.1)	61.1 (3.9)	68.8 (4.3)	66.3 (3.3)	69.8 (4.6)	<0.05					
EPM	Number of entry to closed arm	8.4 (1.1)	9.0 (1.1)	11.1 (0.9)	9.7 (1.1)	8.9 (1.0)	10.4 (0.8)	10.4 (1.0)	10.5 (1.1)						
	Ratio of time spent in open arm (%)	10.3 (3.1)	8.0 (1.9)	7.0 (1.5)	5.1 (1.7)	4.2 (2.2)	4.0 (1.3)	3.2 (1.0)	3.5 (1.0)						
	Ratio of entering number to open arm (%)	21.8 (3.3)	19.1 (3.3)	15.3 (2.8)	9.9 (2.9)	9.3 (3.3)	9.7 (2.4)	5.7 (1.8)	7.8 (2.1)	<0.05					
PAL	Latency to cross in conditioning trial (s)	4.7 (0.4)	5.2 (0.6)	5.9 (0.6)	6.0 (0.4)	7.0 (0.6)	5.5 (0.4)	5.5 (0.5)	5.7 (0.5)						
	Latency to cross in test trial (s)	122.9 (17.9)	144.1 (14.2)	156.9 (12.4)	173.2 (6.6)	148.3 (14.0)	164.6 (8.8)	152.1 (11.6)	127.4 (18.0)	<0.05					

*Values in each group represent mean (SE). p-values are derived from Two-Way ANOVAs (mother treatment × offspring treatment). AGD, anogenital distance; OF, open field; EPM, elevated plus-maze; PAL, passive avoidance learning*.

Significant main effects of *in utero* DES exposure, regardless of foster mother exposure, were also observed. Female offspring exposed to DES *in utero* exhibited a decreased total number of transitions in the OF compared to that of oil-exposed offspring regardless of foster mother treatment (oil-pups: 141.5 ± 5.6, des-pups: 125.6 ± 4.4, means of pooled data by pup treatments); [*F*_(1/66)_ = 6.52, *p* < 0.05]. In contrast, OF transition number tended to increase in DES-exposed male offspring compared to that in oil-exposed male offspring [oil-pups: 130.5 ± 5.1, des-pups: 138.6 ± 4.0; *F*_(1/69)_ = 2.88, *p* = 0.098] regardless of foster mother treatment, although the difference did not reach statistical significance.

**Figure 1:**
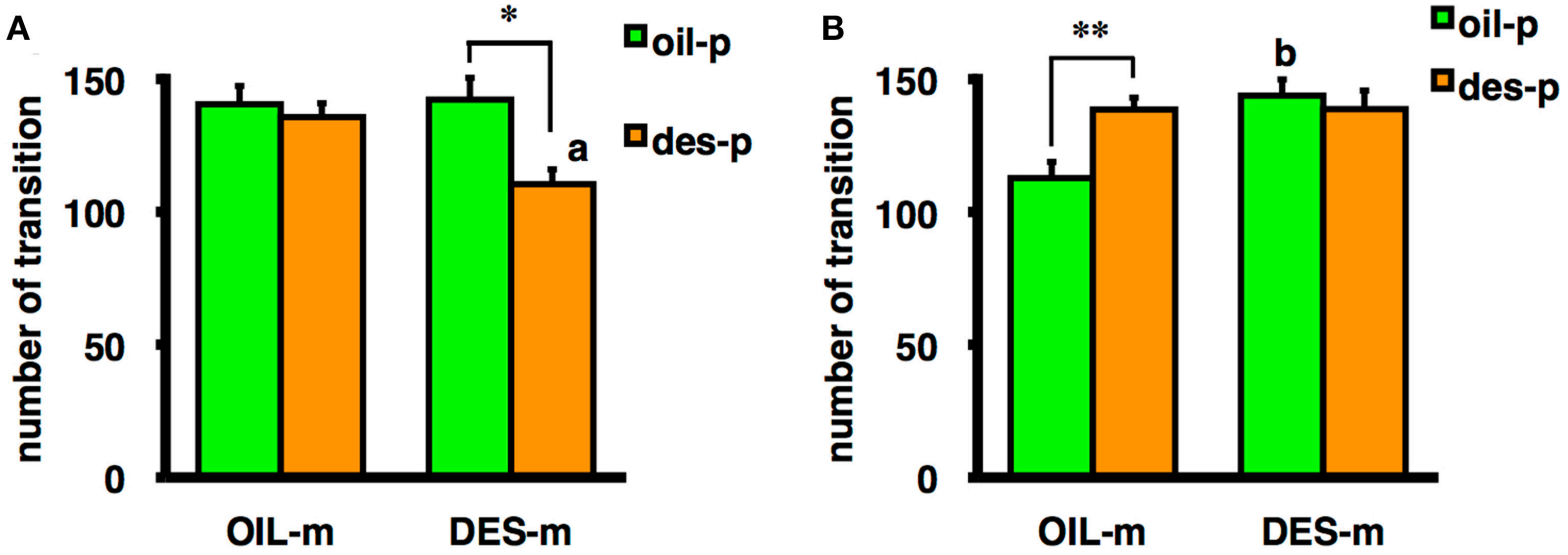
**Mean number of transitions during the OF test by female (A) and male (B) offspring prenatally exposed to DES (des-p group) or oil vehicle (oil-p group), then reared by DES-exposed foster mothers (DES-m) or oil-treated foster mothers (OIL-m)**. Significant difference between groups (^*^p < 0.05, ^**^p < 0.01). Letter above the bar indicates the difference vs. the control group receiving the same prenatal treatment (^a^*p* < 0.01, ^b^*p* < 0.01). Statistical differences were confirmed by the tests of simple main effect.

Moreover, the interaction between mother treatment and offspring treatment was significant for the number of transitions in the OF for male offspring [*F*_(1/69)_ = 6.41, *p* < 0.05] and nearly significant for female offspring [*F*_(1/66)_ = 3.53, *p* = 0.065]. A test of a simple main effect demonstrated that male offspring not exposed to DES *in utero* but reared by DES-exposed mothers exhibited a significantly greater number of transitions in the OF than male offspring not exposed to DES *in utero* and reared by OIL-treated mothers [*F*_(1/69)_ = 12.37, *p* < 0.01] (Figure 1B), indicating that aberrant maternal behavior (from DES exposure) can influence male offspring behavior without *in utero* DES exposure. Female offspring exposed to DES *in utero* and reared by DES-exposed foster mothers tended to exhibit fewer transitions than female offspring exposed to DES *in utero* and reared by OIL-treated foster mothers [*F*_(1/66)_ = 6.187, *p* < 0.05] (Figure 1A); thus, suggesting a role for aberrant maternal behavior. In contrast, the number of OF transitions by male offspring exposed to DES *in utero* and reared by oil-treated foster mothers was significantly higher than for males treated with oil *in utero* and reared by oil-treated foster mothers [*F*_(1/69)_ = 8.73, *p* < 0.01] (Figure 1B). Finally, female offspring exposed to DES *in utero* and reared by DES-exposed foster mothers tended to exhibit fewer transitions than female offspring treated with OIL *in utero* and reared by DES-exposed foster mothers [*F*_(1/66)_ = 9.83, *p* < 0.01] (Figure 1A). These results indicate that the effects of DES exposure on offspring behavior are dependent on both sex and postnatal rearing.

On the other hand, DES exposure both of the foster mother and of the offspring did not influence body weight or anogenital distance at weaning in either male or female offspring.

## Discussion

To the best of our knowledge, this is the first study to demonstrate that endocrine disruption in pregnant mice by DES exposure alters maternal behavior. And the results in this study suggest that these alterations in maternal behavior may impact offspring behavior independent of DES exposure *in utero*. Indeed, male offspring reared by DES-exposed foster mothers showed higher activity in the OF test irrespective of DES exposure *in utero*, whereas female offspring reared by DES-exposed foster mothers showed greater fear and anxiety-like behaviors as indicated by longer mean latency to enter the shock chamber in the PAL test, a lower ratio of open arm entries in the EPM, and less time spent in the OF center area (Table 2), irrespective of DES exposure *in utero*. The enhanced latency to entering in the test trial of PAL in female offspring, which usually indicates improved fear memory, may also reflect trait anxiety as indicated by fewer entries into open arms of the EPM and less time spent in the central area of the OF. On the other hand, the influence of DES-exposure *in utero*, irrespective of exposure to the foster mother, was shown by lower OF activity of female offspring prenatally exposed to DES, whereas the male offspring exposed to DES *in utero* showed slightly higher OF activity (not statistically significant). In summary, DES-exposure of foster mothers decreased locomotion of female offspring in the OF and increased their anxiety-related behavior in the OF and EPM as well as fear memory in the passive avoidance task. At the same time, rearing by DES-exposed foster mothers increased OF locomotion of male offspring. On the other hand, the influence of DES-exposure *in utero* was shown most strongly by female offspring in the OF. These results suggest that the effects of endocrine disruption during pregnancy exert both direct effects on embryos and indirect effects by altering maternal care.

In experiment 2, a significant effect of prenatal DES exposure on the number of transitions in the OF was observed in male offspring reared by a vehicle (OIL)-treated foster mother, indicating a direct effect of DES *in utero* (Figure 1B). Male offspring not exposed to DES but reared by DES-exposed foster mothers also exhibited a significantly greater number of transitions in the OF than male offspring not exposed to DES and reared by OIL-treated mothers, an example of the effect of DES-induced alterations in maternal care (Figure 1B). In contrast, in the females, influence of prenatal DES exposure on the same behavioral index was observed in offspring reared by DES-exposed foster mothers, and postnatal influence of maternal care was observed only in female offspring prenatally exposed to DES (Figure 1A). This result may reflect an interaction between the effects of DES exposure *in utero* and DES-induced deficits in maternal care. In several previous studies, contributions of *in utero* and rearing effects could not be discriminated because the mothers exposed to DES reared their own offspring. In the C57BL/6J strain used in these experiments, general sex differences in OF activity have been reported, with typically higher activity in females (Van Swearingen et al., 2013). Prenatal treatment with an estrogenic endocrine disruptor often diminishes such sex differences (Kubo et al., 2001; Tomihara et al., 2006). Consistent with these previous findings, females prenatally treated with oil and then reared by an OIL-treated foster mother (OIL-oil group) showed a greater number of transitions than males receiving the same treatments. In contrast, DES exposure of both the birth mother and the foster mother (DES-des group offspring) abolished sex differences in OF ambulation. Moreover, preliminary analysis by Three-Way ANOVA (mother exposure × offspring exposure × sex) revealed a significant interaction between sex and both foster mother exposure and offspring exposure [mother exposure × sex: *F*_(1/135)_ = 8.48, *p* < 0.01; offspring exposure × sex: *F*_(1/135)_ = 9.33, *p* < 0.01]. DES exposure of the foster mother enhanced the OF activity of the male offspring, and suppressed that of the female offspring. Furthermore, DES exposure of the offspring enhanced the activity of males and suppressed that of females. These results suggest that the attenuation of sexual differences by DES exposure reported in several studies may result from adding the effects of maternal behavior to the *in utero* effects on sexual development of the offspring.

The maternal effects on offspring OF activity were both sex and treatment dependent. Rearing by a DES-exposed mother enhanced the OF activity of male offspring from OIL-treated birth mothers but decreased the OF activity of female offspring from DES-exposed birth mothers. Several studies reported that the influences of maternal loss were more severe in male than female mice (Kikusui et al., 2006, 2013). This may explain why maternal effects were observed in female offspring prenatally exposed to DES but not in DES-exposed males. Females may be masculinized and the males feminized by DES exposure *in utero*; therefore, the OF activity of DES-exposed female offspring was more sensitive to maternal care.

The present study suggests that endocrine disruption during pregnancy interferes with critical behavioral adaptations of the mother and hence the normal behavioral development of pups. A few previous studies reported a decline in maternal behavior following exposure to the estrogenic endocrine disruptor bisphenol A, although this was not the main focus of these studies (Palanza et al., 2002; Kundakovic et al., 2013). A number of mechanisms have been proposed to explain changes in maternal behavior induced by estrogenic agents (Kinsley et al., 2008; Numan and Woodside, 2010). The medial preoptic area (MPOA) is one of the most important neural regions for the regulation of maternal behavior in mammals (Numan, 2006; Numan and Stolzenberg, 2009) because lesions of the MPOA severely disrupt maternal behavior of female rats (Numan, 1974). The expression of c-fos immunoreactive cells in the MPOA of female rats was increased by exposure to pups, and female rats that exhibited maternal behaviors toward pups had more c-fos immunoreactive cells in the MPOA than females that were not maternally responsive (Numan and Numan, 1994; Stafisso-Sandoz et al., 1998). The somal size, number of basal dendritic branches, and cumulative basal dendritic length of MPOA neurons increased in female rats following reproductive experience (Keyser-Marcus et al., 2001). The density of dendritic spines in the hippocampal CA1 region also increased after reproductive experience (Pawluski and Galea, 2006). Increased dendritic spines density in parous females was also found in the amygdala, which has a primary role in emotional reactivity and encoding memories with emotional salience (Pessoa and Adolphs, 2010; Pare and Duvarci, 2012). All of these areas richly express estrogen receptors (Mitra et al., 2003), and administration of estradiol and progesterone to naïve female rats induced neuronal changes resembling those observed in parous females (Keyser-Marcus et al., 2001; Kinsley et al., 2006). These results suggest that DES prevents the neuroplastic changes in these areas associated with reproductive experience through abnormal activation of estrogen receptors. To confirm this hypothesis, future studies should examine neuronal health, dendritic morphology, gene expression patterns, and synaptic plasticity in the hippocampus, amygdala, and MPOA following DES exposure during gestation. In addition, because the estrogenic regulation of maternal behavior is thought to be mediated by prolactin and oxytocin activity (Bridges et al., 1985; McCarthy, 1995), the role of these neuropeptides in these areas should be examined.

We cannot eliminate a contribution of stress from transportation and oral treatment during pregnancy. Many studies have demonstrated that stress during pregnancy impairs maternal mental health (Smith et al., 2004; Hillerer et al., 2012). In addition, it was suggested that gavage itself could affect gene expression in the brains of the offspring (Cao et al., 2013), though we made efforts to reduce maternal stress by avoiding direct insertion of the administration needle into the stomach. These methodological factors were not thought to be critical for estimation of the influence of prenatal DES exposure because all dams (DES- and OIL-treated) were exposed to the same level of stress including transportation and oral administration during gestation. Actually, several previous studies on DES effects used dams transported during pregnancy and the oral administration method (e.g., Cummings et al., 1999; Tanaka et al., 2004; Fujimoto et al., 2013). Even if the stress limits any differences between DES- and OIL-treated animals, our results demonstrated that DES exposure during pregnancy appeared to affect specific behavioral traits of mother mice and their offspring, at least in some situations. An estimation of the interaction between endocrine disruption and stress during pregnancy was beyond the scope of this study but should be examined in future studies.

In conclusion, the results of this study indicate that endocrine disruption by DES exposure during pregnancy disrupts the adaptive behavioral changes in dams and that these behavior alterations in turn can impact pup behavior independent of DES exposure *in utero*. These findings underscore the risk of environmental endocrine disruptors to both the mother and fetus. Further studies on the influence of endocrine disruptors on maternal behavior induced by reproductive experience may lead to a better understanding of the mechanisms underlying developmental impairments and facilitate interventions for reducing the risks conferred by these agents on both pregnant women and offspring.

### Conflict of interest statement

The authors declare that the research was conducted in the absence of any commercial or financial relationships that could be construed as a potential conflict of interest.
